# A novel *BRCA1* splicing variant detected in an early onset triple-negative breast cancer patient additionally carrying a pathogenic variant in *ATM*: A case report

**DOI:** 10.3389/fonc.2023.1102184

**Published:** 2023-03-21

**Authors:** Mara Colombo, Patrizia Mondini, Elisa Minenza, Claudia Foglia, Annamaria Mosconi, Carmen Molica, Lorenza Pistola, Vienna Ludovini, Paolo Radice

**Affiliations:** ^1^ Unit of Molecular Bases of Genetic Risk and Genetic Testing, Department of Experimental Oncology, Fondazione IRCCS Istituto Nazionale dei Tumori, Milan, Italy; ^2^ Department of Medical Oncology, Santa Maria della Misericordia Hospital, Perugia, Italy

**Keywords:** *BRCA1*, *ATM*, double heterozygote, spliceogenic variant, case report

## Abstract

The widespread adoption of gene panel testing for cancer predisposition is leading to the identification of an increasing number of individuals with clinically relevant allelic variants in two or more genes. The potential combined effect of these variants on cancer risks is mostly unknown, posing a serious problem for genetic counseling in these individuals and their relatives, in whom the variants may segregate singly or in combination. We report a female patient who developed triple-negative high grade carcinoma in the right breast at the age of 36 years. The patient underwent bilateral mastectomy followed by combined immunotherapy and chemotherapy (IMpassion030 clinical trial). Two years later she developed a skin recurrence on the right anterior chest wall. Despite intensive treatment, the patient died at 40-year-old due to disease progression. Gene panel testing of patient’s DNA revealed the presence of a protein truncating variant in *ATM* [c.1672G>T; p.(Gly558Ter)] and of a not previously reported variant in the *BRCA1* exon 22 donor splice site [c.5406+6T>G], whose clinical significance was unknown. The analysis of patient’s RNA revealed the up-regulation of two alternative *BRCA1* mRNA isoforms derived from skipping of exon 22 and of exons 22-23. The corresponding predicted protein products, p.(Asp1778GlyfsTer27) and p.(Asp1778_His1822del) are both expected to affect the BRCA1 C Terminus (BRCT) domain. The two variants were observed to co-occur also in the proband’s brother who, in addition, was heterozygous for a common variant (c.4837A>G) mapped to *BRCA1* exon 16. This allowed to ascertain, by transcript-specific amplification, the lack of functional mRNA isoforms expressed by the c.5406+6T>G allele and provided evidence to classify the *BRCA1* variant as pathogenic, according to the guidelines of the Evidence-based Network for the Interpretation of Germline Mutant Alleles (ENIGMA) consortium. To our knowledge, excluding two cases detected following the screening of population specific recurrent variants, only one *ATM*/*BRCA1* double heterozygote has been reported in the literature, being the case here described the one with the youngest age at cancer onset. The systematic collection of cases with pathogenic variants in more than one cancer predisposition gene is needed to verify if they deserve *ad hoc* counseling and clinical management.

## Introduction

Germline pathogenic variants in the *BRCA1* (MIM# 113705) and *BRCA2* (MIM# 600185) genes are the main risk factors for hereditary breast and ovarian cancer (HBOC). For several years, following their identification in the 1990s (1, 2), genetic tests in HBOC patients were limited to the screening of these two genes, using different mutation analysis techniques, including direct Sanger sequencing. However, it was soon apparent that only a fraction (approximately 15-25%) of HBOC families fulfilling the criteria for clinical testing carry pathogenic variants of BRCA genes (3, 4). Moreover, approximately 10-20% of tests detects the presence of variants, termed variants of uncertain significance (VUS), whose effect on cancer risk is unknown (5). More recently, other breast cancer (BC) and/or ovarian cancer (OC) predisposition genes have been identified (6). The recent advent of Next Generation Sequencing (NGS), in addition to minimizing the costs and time of genetic analyses, has enabled the simultaneous screening of multiple genes. Therefore, NGS has implemented the potential for the detection of pathogenic variants in HBOC genes other than *BRCA1* and *BRCA2* and for the identification of subjects with pathogenic variants in more than one cancer predisposition gene. In fact, although not frequently, HBOC patients have been described with double heterozygous pathogenic variants in *ATM*, *BRCA1*, *BRCA2*, *CHEK2*, *MSH6*, *MUTYH*, *NBN/NBS1* and *RAD50* following gene panel testing (7–13) with a frequency of approximately 0.3% (10, 13). Not surprisingly, double heterozygous carriers are more frequently detected in populations enriched with founder variants (14). To date, the contribute of a double heterozygosity condition for HBOC genes to the severity of the disease and its impact on the genetic counseling and clinical management of the carriers and their relatives is still debated. Further studies are therefore needed to clarify these issues.

In the present study, we report a female patient who developed triple-negative high grade carcinomas in the right breast at the ages of 36 and 38 years. Gene panel testing of patient’s constitutional DNA revealed, in addition to a protein truncating variant in *ATM* [c.1672G>T; p.(Gly558Ter)], the presence of a novel VUS in the consensus sequence of the donor splice site of *BRCA1* exon 22 [c.5406+6T>G]. Gene transcript analysis revealed that the latter affected RNA splicing and allowed its classification as pathogenic. To the best of our knowledge, only three double heterozygotes for *ATM* and *BRCA1* pathogenic variants had been previously reported, being the case here described the one with the youngest age at cancer onset.

## Case presentation

**Figure 1:**
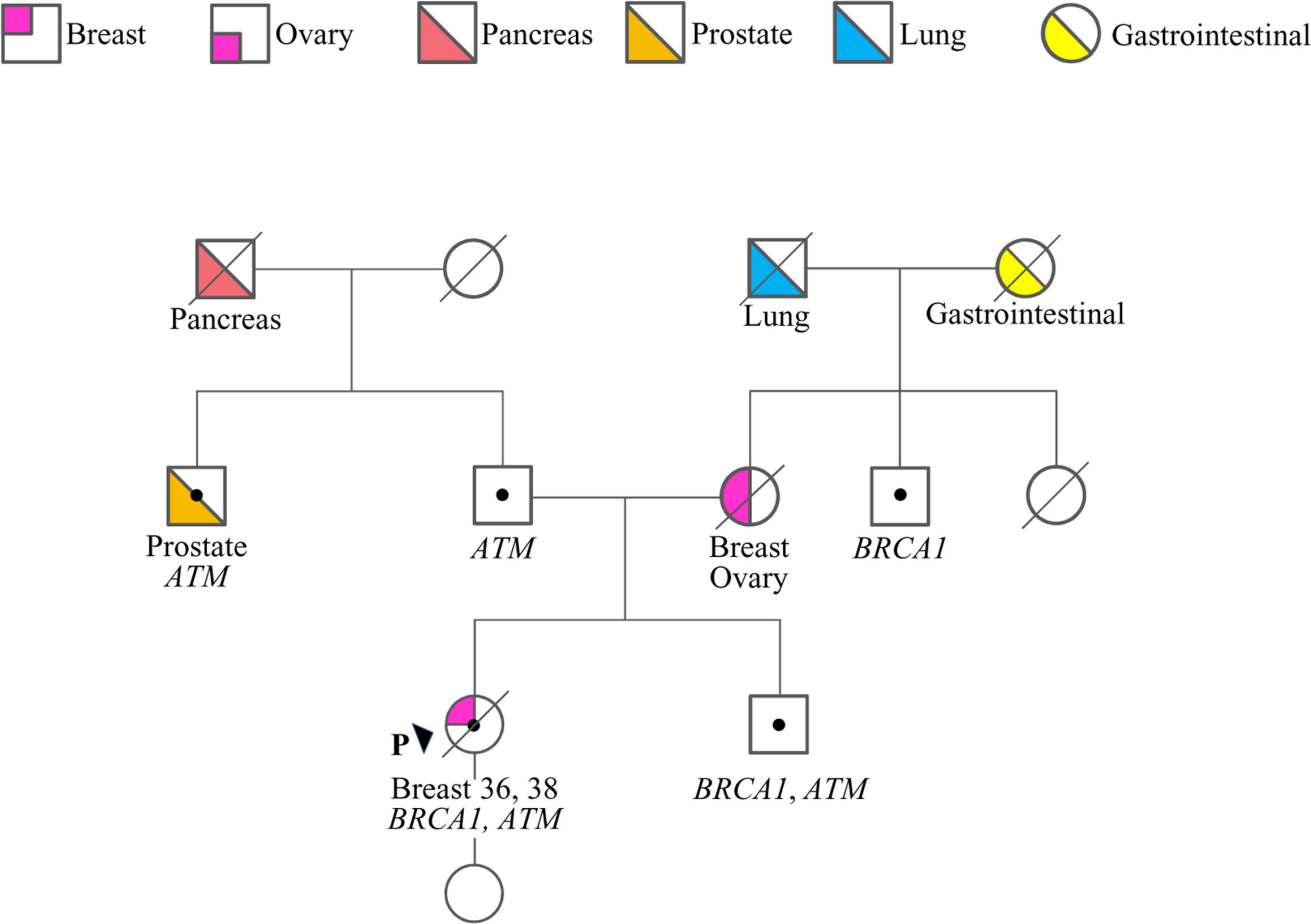
Family pedigree. Males and females are indicated by squares and circles, respectively. The proband (P) is indicated by the arrow. The ages of cancer diagnosis (proband only) and the phenotypes of affected family members are reported under the corresponding symbols, when known. Diagonal slashes indicate deceased individuals. Individuals who underwent genetic analysis are indicated by dots and the genes found mutated are reported.

The case here described (therein termed proband) is a woman who at 36 years old sough genetic counseling due to the cancer history of the mother, who was diagnosed with BC and OC (**Figure 1**). Furthermore, she reported additional cancer cases in second- and third-degree relatives, including lung, gastrointestinal, prostate and pancreatic cancers. Based on family history, the proband was considered eligible for mutation screening of the *BRCA1*, *BRCA2* and *PALB2* genes. An NGS analysis, performed using a small-size panel (Myriapod^®^ NGS BRCA1-2 panel Kit CE-IVD, Diatech Pharmacogenetics), identified the c.5406+6T>G variant in *BRCA1*. At the time of gene testing (April 2018), no data on the clinical significance of this intronic variant were reported in the BRCA Exchange (15) and ClinVar (16) repositories, nor in the literature. A few months later the proband was diagnosed by ultrasound scan with a BC. She opted for a bilateral nipple-areola complex (NAC) sparing mastectomy. A right axillary lymph node dissection was performed for a stage IIB (pT2N1aM0) cancer. The histological examination revealed a triple-negative (ER, PgR, and HER2 negative) invasive ductal G3 carcinoma (ki67 = 50%) with *in situ* component (5%). Considering the diagnosis of BC, the family history and the absence of a definitely pathogenic variant in *BRCA1*, *BRCA2* and *PALB2*, the genetic status of the proband was further investigated with a larger NGS-based panel (Hereditary Cancer Solution (HCS) kit, SOPHiA GENETICS) containing the following 26 genes, *ATM, APC, BARD1, BRCA1, BRCA2, BRIP1, CDH1, CHEK2, EPCAM, FAM175A, MLH1, MRE11A, MSH2, MSH6, MUTYH, NBN, PALB2, PIK3CA, PMS2, PTEN, RAD50, RAD51C, RAD51D, STK11, TP53, XRCC2*. The analysis identified the *ATM* c.1672G>T variant, located in exon 11.

The proband was enrolled in the IMpassion030 clinical trial (NCT03498716) and received adjuvant immunotherapy (atezolizumab) combined with taxan- and anthracyclin-based chemotherapy and with adjuvant radiotherapy (40Gy) for 12 months after diagnosis. Two months after the end of the treatment, the patient underwent prophylactic bilateral hystero-adnexectomy surgery. At 38 years old a skin recurrence on the right anterior chest wall with lymphangitic carcinomatosis was diagnosed consistent with triple-negative BC (ki67 = 90%). She received a combination chemotherapy of carboplatin and gemcitabine for three months as first-line treatment followed by Olaparib 600 mg/day for two months due to disease progression. At the end of the treatment a revaluation computed tomography (CT**)** scan showed skin, lymph node, and pulmonary disease progression. The patient underwent a second line treatment with a combination of oral vinorelbine and capecitabine for four mouths and electrochemotherapy of skin metastases, followed by a further combination chemotherapy with cyclophosphamide-methotrexate-fluorouracil (CMF) for one month. An additional revaluation CT scan showed further disease progression. The patient died at the age of 40 years.

Following targeted genetic testing in the proband’s parents, the c.1672G>T in *ATM* and the c.5406+6T>G in *BRCA1* variants were found to have been inherited from the father and the mother, respectively (**Figure 1**). While the *ATM* variant, which introduces a premature termination codon (PTC) resulting in a non-functional protein [p.(Gly558Ter)], could be considered pathogenic according to the guidelines of the American College of Medical Genetics and Genomics and the Association for Molecular Pathology (ACMG-AMP) (17), the clinical relevance of the *BRCA1* variant remained uncertain.

**Figure 2:**
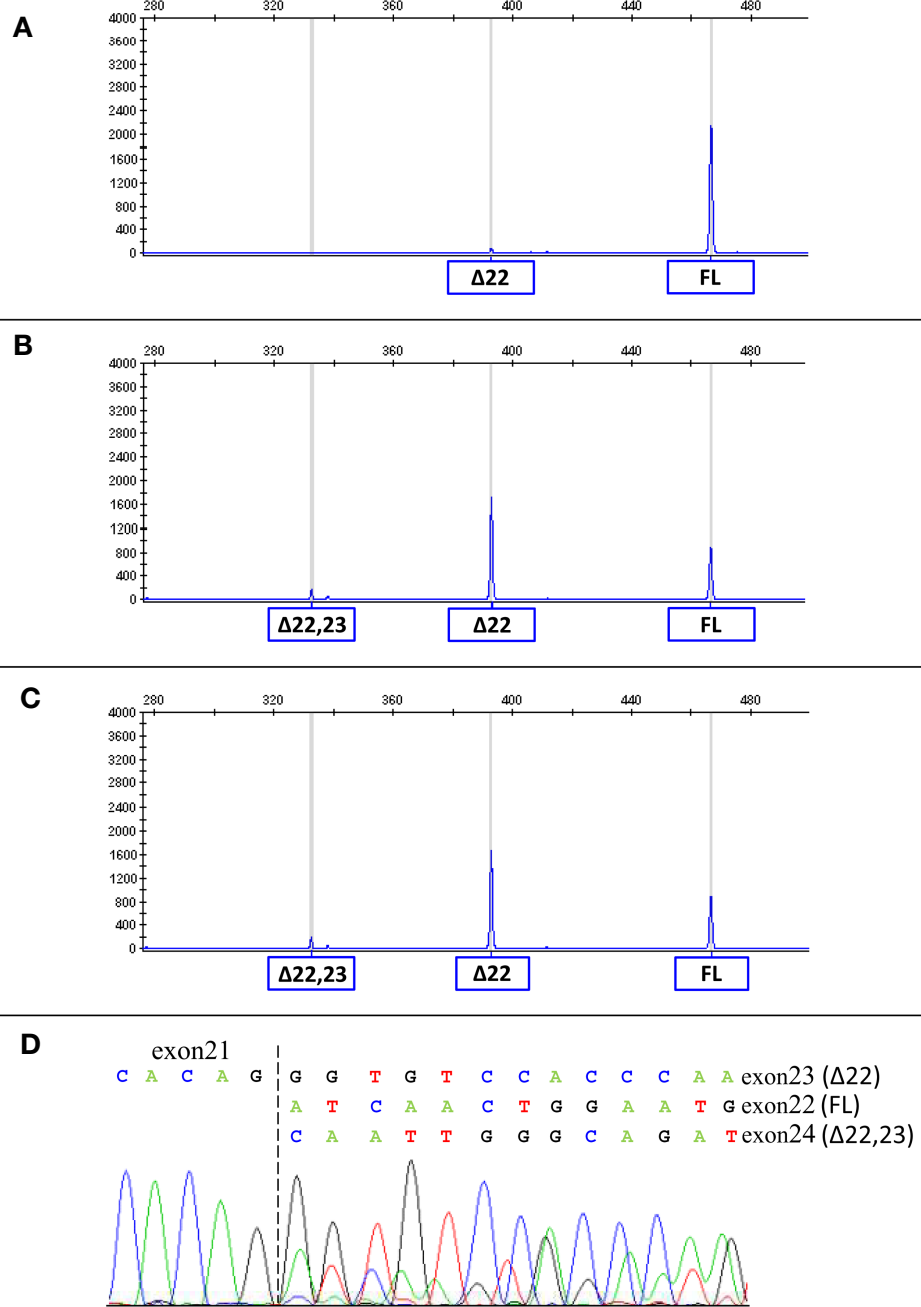
mRNA splicing analysis of the *BRCA1* c.5406+6T>G variant. The capillary electrophoresis patterns of the cDNA fragments spanning *BRCA1* exons 19 to 24 detected in LCLs from a *BRCA1* wild type subject **(A)** and from c.5406+6T>G carriers (proband **(B)** and proband’s brother **(C)**) are shown. The peaks corresponding to normal transcripts maintaining exons 22 and 23 (full-length, FL) and aberrant transcripts (Δ22 and Δ22,23) are indicated. The sequencing of the PCR products **(D)** confirmed the presence of Δ22, Δ22,23 and normal transcripts in carriers of the *BRCA1* c.5406+6T>G variant.

**Figure 3:**
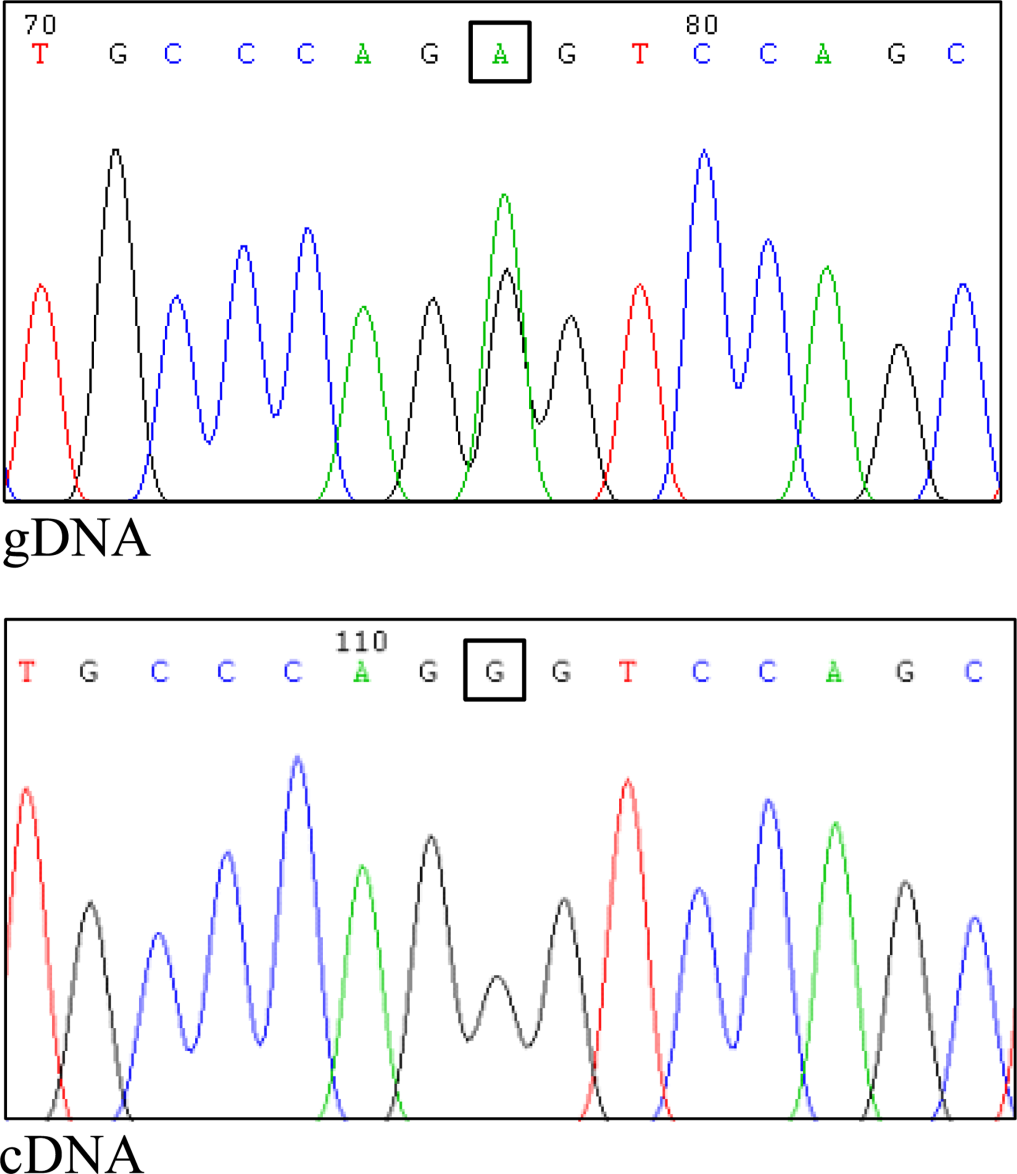
Assessment of allele-specific expression of the normal *BRCA1* transcript. The common variant c.4837A>G, mapped to *BRCA1* exon 16, was proven to be heterozygous in the genomic DNA (gDNA) of the proband’s brother (upper panel). The specific amplification of cDNA fragment maintaining exon 22 (normal transcript) and including the c.4837A>G variant was performed using a forward primer in exon 16 and a reverse primer in exon 22. The sequencing of the RT-PCR product showed a mono-allelic expression of the normal transcript (lower panel), indicating a complete spliceogenic effect of the c.5406+6T>G variant.

Considering that the variant in *BRCA1* is located in the consensus sequence of the donor splice site of exon 22 (according to the Breast Information Core (BIC) nomenclature) (18), a potential effect at mRNA splicing level was investigated by interrogating the Alamut Visual Plus software application (version v1.2.1 | ^©^ 2021 SOPHiA GENETICS). All tools integrated in the software predicted the variant to impact on the splicing by weakening the natural donor site of exon 22. The putative spliceogenic effect was then verified by characterizing the mRNA transcript profile as previously described (19, 20). Briefly, Epstein-Barr virus (EBV)-immortalized lymphoblastoid cell line (LCL) was established from the peripheral blood of the variant carrier. The degradation of transcripts containing premature termination codons (PTCs) *via* nonsense mediated mRNA decay (NMD) was prevented by growing the LCL in the presence of cycloheximide. Cytoplasmic RNA was isolated from the LCL and first strand cDNA was generated for RT-PCR analysis. The primers for the amplification were designed specifically for the variant under study. Furthermore, the reverse primer was labeled with 6-carboxyfluorescein (6-FAM) in order to obtain fluorescent amplification fragments detectable by capillary electrophoresis (CE). The CE profile obtained from the LCL carrying the *BRCA1* variant was compared to that derived from *BRCA1* wild type subjects (reference) and the aberrant products not present in the reference were characterized by Sanger sequencing. Experimental conditions are reported in **Supplementary Table 1**. The analysis revealed the up-regulation of two isoforms derived from the out-of-frame skipping of exon 22 (Δ22, major transcript) and the in-frame skipping of exons 22 and 23 (Δ22,23, minor transcript) (**Figure 2**), both previously described as naturally occurring isoforms (21). The corresponding predicted protein products, p.(Asp1778GlyfsTer27) and p.(Asp1778_His1822del), are both expected to affect the BRCA1 C Terminus (BRCT) of the protein, a clinically relevant functional domain. The *BRCA1* and *ATM* variants were observed to co-occur also in the proband’s brother, unaffected at the time of genetic testing (at age 33 years), who in addition was heterozygous for the common variant c.4837A>G (rs1799966) mapped to *BRCA1* exon 16. PCR fragments spanning the c.4837A>G variant were selectively amplified from the normal transcripts maintaining exon 22 using a reverse primer annealing to this exon and a forward primer annealing to the region upstream of the c.4837A>G variant. The sequence analysis of these amplification products, showing a mono-allelic expression, allowed us to ascertain the lack of functional mRNA isoforms expressed by the allele carrying the c.5406+6T>G variant (**Figure 3**) and to classify the variant as pathogenic (class 5) according to the *BRCA1/2* Gene Variant Classification Criteria of the Evidence-based Network for the Interpretation of Germline Mutant Alleles (ENIGMA) (22). Potential consequences on mRNA splicing were experimentally investigated following the above described approach also for the *ATM* c.1672G>T variant and excluded (data not shown).

## Discussion

In this study, we describe a female patient diagnosed with triple-negative high grade BC, with two heterozygous variants in genes associated with increased risk of BC, namely the pathogenic (protein truncating) variant c.1672G>T in *AT*M and the suspected spliceogenic variant c.5406+6T>G in *BRCA1*. Neither of the two variants had been previously published or reported in publicly available databases. Due to the uncertainty on the clinical relevance of the *BRCA1* variant, we analyzed the LCL RNAs of the proband and her brother, who also carried the same variant. These investigations allowed us to classify the *BRCA1* variant as pathogenic, according to the ENIGMA guidelines. Notably, our observations are consistent with the outcomes of a saturation genome editing (SGE) assay, which were published after the identification of the variant in the case here described, that suggested a loss-of-function effect of the *BRCA1* c.5406+6T>G variant (23).

To the best of our knowledge, only three double heterozygotes for germline pathogenic variants in the *BRCA1* and *ATM* genes had been previously reported. Notably, two cases, both presenting the c.181T>G [p.(Cys61Gly)] variant in *BRCA1* and the c.5932G>T [p.(Glu1978Ter)] variant in *ATM*, had been detected following targeted screening of population-specific pathogenic variants (24). Conversely, the third case, carrying the c.5123C>A [p.(Ala1708Glu)] variant in *BRCA1* and c.2413C>T [p.(Arg805Ter)] variant in *ATM* was identified following a gene panel NGS analysis (7). The latter case reported multiple cancer diagnoses in addition to breast cancer, including an infiltrating carcinoma of the ampulla and a clear-cell endometrial adenocarcinoma.

Both the *ATM* and *BRCA1* genes code for key proteins involved in DNA double-strand breaks (DSBs) repair by homologous recombination (HR), a crucial mechanism for maintaining genomic integrity and preventing cancer development (25). It is debated whether, given the involvement of the two proteins in the same pathway, the risk of cancer and the severity of the disease can be increased in carriers of pathogenic variants in both genes, compared to single pathogenic variant carriers. Notably, it has been observed that in *Brca1*-null murine mammary tissue the heterozygous loss of *Atm* affects both mammary development, reducing ductal branching during puberty, and tumor severity, increasing invasiveness and causing undifferentiated tumor types, a phenotype not observed in the presence of *Atm* wild-type alleles (26). A later study demonstrated that in murine thymocytes the hemizygosity for both *Atm* and *Brca1* decreases radiation-induced apoptosis compared to single *Atm* or *Brca1* hemizygous cells (27). Finally, it has been observed that, compared to single *Atm* and *Brca1* heterozygous cells, mouse embryo fibroblasts double mutated in *Atm* and *Brca1* show an increased frequency of neoplastic transformation, a higher genomic instability, a delayed recognition of DNA damage induced by photon irradiation and an incomplete DNA repair (28).

Loss of function (LOF) *BRCA1* variants are associated with a high chance of cancer, with estimated cumulative risks by age 80 years of 72% and 44% for breast and ovarian cancer, respectively (29). Conversely, LOF *ATM* variants are considered moderate risk factors for breast cancer (cumulative risk by age 80 years of 27%) (30), with some limited evidence of association with ovarian cancer risk (31). To date, several common genetic variants, conferring a modest increase of breast/ovarian cancer risk in the general population, have been ascertained to modulate the penetrance of *BRCA1* pathogenic variants (32). It is conceivable that the same modifier effect could be elicited by rare pathogenic variants in genes associated with moderate cancer risk. Consistent with this hypothesis, it has been recently reported that the occurrence of additional truncating variants in DNA repair genes might lead to an earlier onset of breast cancer in carriers of pathogenic *BRCA1* variants (33). However, in the four *BRCA1*/*ATM* double heterozygotes reported to date (7, 24 and present study), the age of first breast cancer diagnosis ranged from 36 to 55 years (the patient here described being the one with the earliest age of onset) with a median age of 43,2 years, similar to that (42 years) observed in carriers of single pathogenic variants in *BRCA1* (34). Analogously, when considering the tumor pathological characteristics, no specific phenotypes were observed in double heterozygous carriers compared to *BRCA1* pathogenic variant carriers. In all four cases the diagnosed breast cancers were estrogen receptor negative and in two of them (the patient described by Andres et al. (7) and our patient) they were classified as high grade triple-negative carcinomas, which are usually associated with an unfavorable prognosis. However, they are also the more represented breast cancer subtype associated with *BRCA1* pathogenic variants, being detected in approximately 65% of carriers with a breast cancer diagnosis overall (35). Conversely, the observation that both our proband and the case described by Andres et al. (7) developed multiple malignancies is in favor of a particularly aggressive clinical phenotype possible associated with the simultaneous occurrence of *BRCA1* and *ATM* pathogenic variants in the same individual. Based on genetic status and family history, the proband’s healthy brother, carrying both *BRCA1* and *ATM* pathogenic variants, was recommended to undergo intensive surveillance, including endoscopic examination of the lower gastrointestinal tract every five years, yearly screening including mammography with breast magnetic resonance imaging (MRI), abdominal ultrasound scan and prostate specific antigen (PSA) test, starting from the age of 40 years.

In conclusion, the number of carriers of constitutional pathogenic variants in both *BRCA1* and *ATM* reported to date are too limited to assess if their cancer risks differ from those of individuals carrying a single *BRCA1* pathogenic variant. Therefore, the systematic collection of these cases is needed to address this issue and to define if, due to their particular genetic condition, they deserve *ad hoc* counseling and clinical management, including specific risk reduction measures.

## Data availability statement

The original contributions presented in the study are included in the article/**Supplementary Materials**. Further inquiries can be directed to the corresponding author.

## Ethics statement

The studies involving human participants were reviewed and approved by Ethics Committee of the Santa Maria della Misericordia Hospital. The patients/participants provided their written informed consent to participate in this study. Written informed consent was obtained from the individual(s) for the publication of any potentially identifiable images or data included in this article.

## Author contributions

MC conceived the study, designed and performed the *in silico* and *in vitro* splicing analyses, and drafted the manuscript. VL contributed to conception of the study. EM, AM, and CM treated and followed the patient and her family. LP and VL performed the NGS analysis. PM provided technical assistance to the *in vitro* splicing experiments. CF established the LCLs. VL and EM contributed to the draft. PR made substantial contributions to conception of the study and reviewed the manuscript. All authors contributed to the article and approved the submitted version.
