# Corrigendum: A novel *BRCA1* splicing variant detected in an early onset triple-negative breast cancer patient additionally carrying a pathogenic variant in *ATM*: a case report

**DOI:** 10.3389/fonc.2023.1306238

**Published:** 2023-10-23

**Authors:** Mara Colombo, Patrizia Mondini, Elisa Minenza, Claudia Foglia, Annamaria Mosconi, Carmen Molica, Lorenza Pistola, Vienna Ludovini, Paolo Radice

**Affiliations:** ^1^ Unit of Molecular Bases of Genetic Risk and Genetic Testing, Department of Experimental Oncology, Fondazione IRCCS Istituto Nazionale dei Tumori, Milan, Italy; ^2^ Department of Medical Oncology, Santa Maria della Misericordia Hospital, Perugia, Italy

**Keywords:** *BRCA1*, *ATM*, double heterozygote, spliceogenic variant, case report

In the published article, the investigated *BRCA1* variant was incorrectly reported as c.5406+6T>C. The correct annotation of the variant is c.5406+6T>G.

The authors apologize for this error and state that this does not change the scientific conclusions of the article in any way. The original article has been updated.

