# Supplementary material for: A novel BRCA1 splicing variant detected in an early onset triple-negative breast cancer patient additionally carrying a pathogenic variant in ATM: A case report
Source: Front Oncol. 2023 Mar 21;13:1102184. doi: 10.3389/fonc.2023.1102184 (PMC10072264; doi:10.3389/fonc.2023.1102184)
Supplement: Supplementary file 1 [file Table_1.docx]

**Supplementary Table 1. Experimental conditions for mRNA splicing analyses**

| **ANALYSIS** | **GENE** | **VARIANT** | **FORWARD PRIMER** | **EXON** | **REVERSE PRIMER** | **EXON** | **T_a_ ^b^** | **# CYCLES ^c^** |
| --- | --- | --- | --- | --- | --- | --- | --- | --- |
| RT-PCR and capillary electrophoresis |  | | | | | | | |
|  | *BRCA1* | c.5406+6T>C | 5'- GGGTGACCCAGTCTATTAAAG-3' | 19 | 5'- CTGTGGCTCTGTACCTGTG -3' ^a^ | 24 (UTR) | 62°C | 30 |
| RT-PCR and sequencing |  | | | | | | | |
|  | *BRCA1* | c.5406+6T>C | 5'- gaagtcagaggagatgtgg -3' | 20 | 5'- GGTAGAGTGCTACACTGTCC -3' | 24 | 58°C | 35 |
|  | *BRCA1* | c.4837A>G | 5'- gatccttctgaagacagagc -3' | 16 | 5'- CACCACAGAAGCACCACAC-3' | 22 | 58°C | 35 |
|  | *ATM* | c.1672G>T | 5'-GCCTTACGGAAGTTGCATTG -3' | 10 | 3'- GAGAAGCCAATACTGGACTG-5' | 13 | 58°C | 35 |

^a^: The primer was labeled with 6-carboxyfluorescein (6-FAM) at the 5'-end.

^b^: T_a,_ annealing temperature of experimental protocols.

^c^: number of amplification cycles in the RT-PCRs.
